# Mental Illness Stigma in Black, Latina/o, and Asian Americans

**DOI:** 10.1007/s40615-024-02259-8

**Published:** 2024-12-18

**Authors:** Andrew M. Subica, Bruce G. Link

**Affiliations:** 1 Riverside School of Medicine, Department of Social Medicine, Population, and Public Health, University of California, 900 University Ave, Riverside, CA 92521, U.S.A.; 2 Riverside School of Public Policy, University of California, 900 University Ave, Riverside, CA 92521, U.S.A.

**Keywords:** Stigma, mental health help-seeking, discrimination, racial minorities

## Abstract

Mental illness stigma has significant psychiatric consequences and can impede mental health treatment seeking, especially among racial minority groups; who are understudied in stigma research and experience striking treatment disparities. Guided by a novel empirical model of racial minority stigma and treatment seeking, this study investigated stigma and its effects on treatment seeking in Black, Latina/o, and Asian American adults. Data were collected via national panel survey from 613 Black, Latina/o, and Asian American adults. Perceptions of mental illness including seriousness, treatability, causal attributions, desired social distancing, and perceived dangerousness were assessed. Data were analyzed and compared with a nationally representative sample of the U.S. public from the 2018 General Social Survey. Minority participants exhibited stronger mental illness stigma than the U.S. public, with Black, Latina/o, and Asian American participants largely perceiving mental illness as less serious, less treatable, and desiring greater social distance from individuals with major depression, who were perceived as potentially dangerous. Notably, different stigma components significantly associated with willingness to seek treatment differently across Black, Latina/o, and Asian American participants. Overall, study findings indicate that mental illness stigma is strong and associates with treatment seeking in Black, Latina/o, and Asian Americans, suggesting a need to develop culturally tailored interventions to reduce stigma and associated treatment utilization disparities in these underserved minority groups.

## Introduction

Mental illness stigma is the process by which individuals with mental disorders are labeled, stereotyped, and discriminated against by the general public [1, 2]; often for the purposes of exploiting, dominating, or excluding them from society [3]. Through this process, stigma affects individuals’ psychological health and psychosocial functioning by exposing them to numerous social and economic consequences including loss of social status, education/employment opportunities, and experiencing of hostility, marginalization, and ostracization [1, 4]. Thus, stigma is theorized to impede mental health treatment seeking as the perceived threat of shaming, prejudice, and discrimination from one’s social environments may discourage individuals from seeking help [1, 5, 6]. Consequently, stigma is considered the leading barrier to treatment seeking by many experts [1, 7–9]. This includes among racial minorities, who are understudied in stigma research [10–12] and experience strong treatment disparities [10, 13–16].

Although stigma is often perceived to occur largely within the individual (e.g., as internalized stigmatizing beliefs/attitudes), decades of research have revealed that stigma is heavily socially constructed and enforced [6] with the social environment: (1) providing the context in which discrimination is anticipated and experienced by individuals, and (2) defining which personal attributes (e.g., mental illness) are devalued/rejected by others [3, 17]. In this way, individuals considering mental health treatment are likely to closely appraise the prevailing stigma beliefs and attitudes of their social environments (i.e., “stigma contexts”) to identify potential threats to their social status and well-being [1, 11, 18]. In highly stigmatizing contexts, individuals frequently become aware of and adopt negative societal beliefs, attitudes, and intentions toward mental disorders and treatment seeking [19–21]. Accordingly, we postulate that individuals embedded in strong stigma contexts—in regards to how their affiliate groups negatively perceive and treat persons with mental disorders—will be hesitant to seek formal treatment for mental illness due to the public risks and self-stigma associated with treatment seeking in these contexts [22–24]. Confirming the public risks, data from the General Social Survey (GSS)—the primary U.S. study assessing stigma—uncovered high levels of mental illness stigma in the U.S. public. For example, in 2018, 79% and 67% of U.S. adults were unwilling to work closely with or have an individual with schizophrenia marry into their family, respectively [25].

For racial minority groups, the influence of stigma on treatment seeking may be even more pronounced as studies indicate minorities seek mental health treatment at lower rates than the general public [10, 13–16]. Although several reasons for this treatment disparity exist including elevated rates of uninsurance/underinsurance, decreased access to mental health care, lack of culturally responsive providers/services, and cultural mistrust [26, 27], stigma is suspected to play a critical role in curtailing minority treatment seeking [11].

Yet, to our knowledge, empirical data describing mental illness stigma in U.S. racial minority groups remains largely unavailable. Notably, although the GSS [25, 28] is the leading source of data describing the U.S. public’s stigma beliefs/attitudes for decades, its design has never included sufficiently diverse populations to: (1) permit analysis within and between racial minority groups, or (2) draw comparisons to the U.S. public. For instance, in a recent study detailing U.S. stigma using aggregated 1996–2018 GSS data, Black and Latina/o individuals were collapsed into an opaque “non-White category” to avoid estimation problems [25].

Consequently, surprisingly little is known about the nature and context of mental illness stigma in disaggregated racial minority groups, producing two empirical gaps. First, we do not know whether stigma contexts differ across the largest U.S. racial minority groups in ways that impact their treatment seeking, and thus may contribute to their treatment disparities. Second, it is unclear which components of these stigma contexts most affect treatment seeking in these groups; preventing researchers from discerning whether tailored interventions vs. a one-size-fits-all approach should be applied to reduce stigma in minority communities. Using a nationwide survey panel, we sought to address these gaps by conducting a detailed investigation of mental illness stigma in Black, Latina/o, and Asian American adults.

### Stigma Context Model

**Fig. 1:**
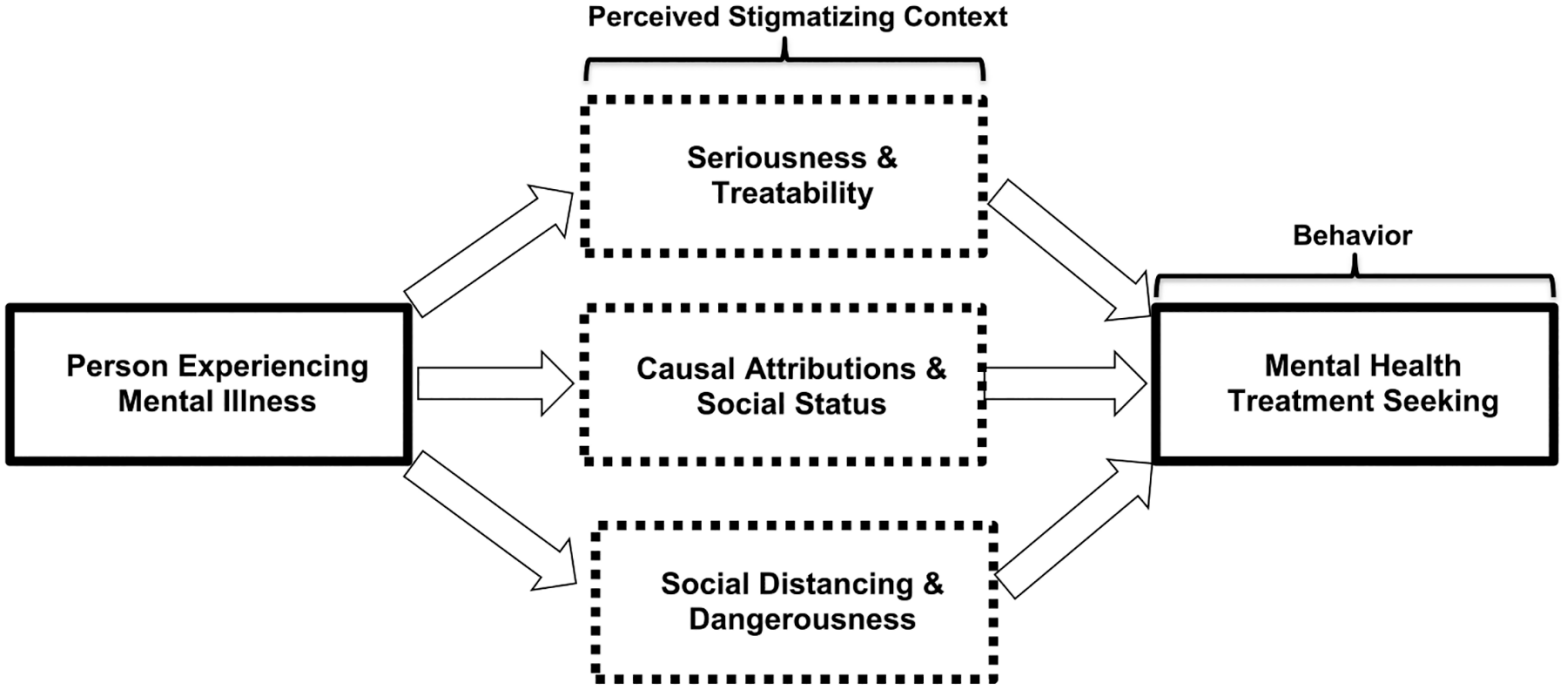
Minority Stigma Context and Treatment Seeking Model

Undergirding this investigation, we integrated key concepts/theories from the stigma and treatment-seeking literatures to develop a novel empirical model of racial minority stigma and treatment seeking. Depicted in Fig. 1, our model proposes that individuals living in unfavorable stigma contexts for treatment seeking might exhibit stigmatizing patterns of beliefs/attitudes in at least one of three empirically-derived domains: (1) “Seriousness and Treatability,” (2) “Attributions and Social Status,” and (3) “Social Distance and Dangerousness.”

In the “Seriousness and Treatability” domain—drawing from the Health Belief Model and minority help-seeking models [29–31]—individuals living in a restrictive treatment-seeking context would perceive mental disorders as: (1) not serious problems, (2) likely to improve on its own, and (3) unlikely to improve with treatment. Under “Attributions and Social Status,” individuals would be less likely to attribute mental disorders to neurobiological causes (e.g., genetics, brain disease) vs. sociomoral ones (e.g., character failings, negative upbringing) as prior research indicates neurobiological attributions may be more supportive of treatment seeking than other attributions [32–34]. Because stigma is strongly linked to prejudice/discrimination—such that some scholars consider them overlapping constructs [3] whose social and psychological consequences may provoke worse outcomes than the inciting mental disorder [35–37]—individuals in a restrictive stigma context would also judge individuals with mental disorders as justly possessing lower social status than, and being inferior to, individuals without mental disorders. Under “Social Distance and Dangerousness,” individuals would perceive persons with mental disorders as potentially dangerous and desire social distance from them as perceived dangerousness is a pervasive mental illness stereotype and social distancing a common response [28, 38, 39] believed to comprise stigma’s “backbone” [40].

### Study Purpose

Guided by our empirical model, this paper sought to characterize mental illness stigma in Black, Latina/o, and Asian American adults by assessing their perceptions of the: (1) seriousness and treatability of major depression and schizophrenia, (2) causes of these disorders and perceived impact on social status, and (3) desired social distance from, and dangerousness of, individuals with these disorders. We assessed Black, Latina/o, and Asian Americans as they compose the nation’s three largest racial minority groups and there exists minimal stigma data (e.g., from the past decade) about these groups to guide current stigma research/interventions.

Then, to determine whether the stigma contexts of Black, Latina/o, and Asian Americans were more likely to suppress treatment seeking vs. the general U.S. public, we contrasted study data with data from the 2018 GSS: the most current nationally representative survey of U.S. public stigma [25]. Finally, to explore the influence of minority stigma contexts on treatment seeking, we examined which stigma components from our model significantly associated with treatment-seeking willingness—paying special attention to differences between our minority groups.

Based on prior research, we postulated that we would find possible differences in the stigma contexts/patterns of Black, Latina/o, and Asian American participants as (1) culture has been shown to strongly shape the meanings and outcomes of stigma across different racial groups, with (2) each minority group possessing distinct cultural values, practices, and experiences that may uniquely influence their patterns of stigma and treatment-seeking behaviors [6, 41, 42]. As an example of potential differences in stigma contexts that may emerge between minority groups, because a major theme for Black Americans within the stigma research is the central influence of historic racism and discrimination on their stigma experiences—which in turn may not be as central to the cultural/stigma experiences of our other minority groups [41]—we may expect Black participants to display some differences in their stigma context from Latina/o and Asian American participants in the study.

## Materials and Methods

### Study Design

Following approval by the University of California, Riverside Institutional Review Board, data were collected via Qualtrics Research Panels from 614 U.S.-dwelling adults, resulting in stigma data from a national sample of 188 Black, 198 Latina/o, and 186 Asian American participants from across the U.S. after cleaning. Specifically, participant recruitment was conducted through Qualtrics-recruited survey research panels drawn from multiple data platforms/panels throughout the U.S. using a diverse set of recruitment methods. These methods included advertisements across digital and social media networks, affiliate marketing, and direct e-mail outreach. For instance, for the e-mail outreach, the Qualtrics team recruited study participants by sending members of the research panels direct e-mails that included a link to an online screening instrument assessing study eligibility. For all participants across every recruitment method, participants first completed the screening instrument to ensure eligibility according to the sampling parameters created by the study investigators. The sampling parameters were: (1) adults over the age of 18 years old, (2) equivalent numbers of Black, Latina/o, and Asian American participants, (3) equivalent numbers of men and women per group, and (4) random assignment to a race- and gender-matched major depression or schizophrenia vignette from the GSS, described below. By employing multiple recruitment methods across diverse national data platforms/panels, Qualtrics has been confirmed to produce demographically representative data [43] comparable to nationally representative [44, 45] and community-recruited samples [46, 47]. Qualtrics has also proven adept at recruiting representative samples of hard-to-reach populations [48, 49], including racial minorities [50, 51]; the foundation of the present study.

After completing the eligibility screening, within the Qualtrics portal, participants completed and signed the informed consent online and completed the survey, receiving compensation valued between $2-$3. In the survey, Qualtrics included (1) attention checks, and (2) speed checks (measured as half the median survey duration) to ensure high quality responses with participants who failed either quality check screened out of the final dataset by the internal Qualtrics team. Qualtrics then implemented additional data quality reviews to remove low-quality surveys (e.g., inconsistent patterns of responding) in the dataset. Upon receiving the final dataset from the internal Qualtrics team, the investigators further reviewed all data for quality and removed cases following guidelines by Belliveau & Yakovenko [52].

Then, to compare study data vs. the general U.S. public, we incorporated nationally representative 2018 GSS data from the 480 non-institutionalized adults administered the GSS major depression or schizophrenia vignettes. Reinforcing our investigation, the racial composition of the 2018 GSS sample matched earlier GSS samples in being overwhelmingly White (74%; *n* = 354) with smaller Black (15%; *n* = 74), Latina/o (5%; *n* = 25), and Asian (3%; *n* = 14) samples that could not be independently analyzed.

### Measures

Demographics (e.g., age, gender) were collected. Willingness to seek treatment was assessed using the National Comorbidity Survey item [53]: “*If you had a serious emotional problem, would you go for professional help—definitely not go, probably not go, probably go, definitely go*?”.

To determine participants’ stigma context, we adapted the GSS stigma modules [25] by depicting Black, Latina/o, and Asian American men and women with major depressive disorder and schizophrenia; modeling an earlier study with Pacific Islanders [27]. Participants first read their randomly assigned major depressive disorder or schizophrenia vignette—matched to participants’ race and gender—then answered the GSS questions (described below) assessing their beliefs and attitudes about the character.

Seriousness was measured via: “*How serious would you consider [character’s] problem to be—very serious, somewhat serious, not very serious, not serious at all?*” Treatability was assessed by asking how likely the character’s situation would improve (1 = *very unlikely*, 4 = *very likely*): (1) “…*on its own*,” (2) “…*with mental health treatment*,” and (3) “…*with traditional [Afrocentric/Latina/o/Asian] treatments/home remedies?”*.

Attributions were captured by asking participants to rate the likelihood (1 = *very unlikely*, 4 = *very likely*) the character’s situation was caused by: “*major depression/schizophrenia*,” “*chemical imbalance in the brain*,” “*genetic or inherited problem*,” “*own bad character*,” “*way person was raised*,” “*God’s will*,” and “*Life stress*.” “*Neurobiological conception*” and “*sociomoral conception”* were coded as present if participants endorsed: (1) “*chemical imbalance in the brain*” or “*genetic or inherited problem*,” and (2) “*own bad character*” or “*way person was raised*,” as “*likely*” or “*very likely*,” respectively, while “*God’s will”* and “*Life stress*” were coded independently.

To assess perceived social status, we utilized two items asking participants to rate (1 = *very unfair/inaccurate*, 7 = *very fair/accurate*) how fair/accurate they thought it was that individuals without mental disorders: (1) “*have a higher status*,” and (2) “*are superior*” to individuals with mental disorders.

To calculate desired social distance, participants completed the five-item GSS social distance measure [25, 28] that asked participants how willing (1 = *definitely willing*, 4 = *definitely unwilling*) they would be to engage in the following situations with the character: (1) “*move next door*,” (2) “*hang out for a night*,” (3) “*make friends*,” (4) “*work closely on a job*,” and (5) “*marry into your family*.” Item scores were summed and averaged to produce an overall mean distance score (α = 0.73). Perceived dangerousness was measured by asking participants to rate if the character would act violently (1 = *very unlikely*, 4 = *very likely*) toward “*other people*” and “*him/herself*.”

### Statistical Analysis

Analyses were performed in STATA v.18.0. Descriptive statistics analyzed frequencies, means, and standard deviations, with treatment-seeking willingness and stigma variables dichotomized into percentages by combining matched responses (e.g., “*unwilling*” for “*probably unwilling*” and “*definitely unwilling*”; “*likely*” for “*somewhat likely*” and “*very likely*”). To identify significant differences in frequencies of stigma variables between each racial minority group vs. the U.S. public, age-adjusted logistic regressions—incorporating marginal standardization to adjust for the population distribution of confounders across groups/exposures—estimated predicted probabilities for each minority group vs. the GSS sample [54–56].

Then, to test our stigma model we conducted linear regressions of Black, Latina/o, and Asian American participants’ willingness to seek treatment (outcome variable) regressed on the predictors of: (1) demographics, (2) seriousness and treatability, (3) causal attributions (neurobiological conception, sociomoral conception, God’s will), (4) social status (lower status, inferiority), and (5) overall mean social distance and dangerousness (violence toward others, self).

## Results

Sample characteristics are detailed in Table 1. Seventy percent of participants reported willingness to seek treatment including 73% of Black, 68% of Latina/o, and 69% of Asian American participants.

Presented in Table 2, participants in each minority group had lower rates of perceiving major depression and schizophrenia as serious problems than the U.S. public. These differences were significantly lower among Latina/o and Asian American participants for major depression and Black and Latina/o participants for schizophrenia vs. the U.S. public (*p* < 0.05). Significantly higher rates of Latina/o and Asian American participants believed major depression would improve on its own, and Black and Latina/o participants believed schizophrenia would improve on its own vs. the U.S. public (*p* < 0.05). Also, significantly lower rates of Black and Latina/o participants believed treatment would improve major depression, and Black, Latina/o and Asian American participants believed treatment would improve schizophrenia vs. the U.S. public (*p* < 0.05). Majorities of Black, Latina/o, and Asian American participants also believed informal cultural treatments would improve major depression and schizophrenia.

Black and Latina/o participants endorsed neurobiological attributions for major depression and schizophrenia at significantly lower rates than the U.S. public (*p* < 0.05). Black and Latina/o participants also attributed mental illness to God’s will at higher rates than the U.S. public (*p* < 0.05) for both major depression (42% and 36% vs. 14%) and schizophrenia (45% and 42% vs. 18%). Due to these strong endorsement rates, we added God’s will to our regression models.

For social status, over 40% of Black, Latina/o, and Asian American participants reported it was fair that individuals with mental illness had lower social status than those without mental illness. Between 41%−50% of Black, Latina/o, and Asian American participants endorsed individuals with mental illness as inferior to those without mental illness.

Displayed in Table 3, Black, Latina/o, and Asian American participants had significantly higher rates of overall desired social distancing as well as unwillingness to make friends with, socialize, and move next door to individuals with major depression vs. the U.S. public (*p* < 0.05). Toward individuals with schizophrenia, Black and Latina/o participants reported significantly lower rates of overall desired social distancing than the U.S. public (*p* < 0.05) although all minority groups generally displayed equivalent rates of unwillingness to engage in individual social situations as the U.S. public.

For dangerousness, participants in all minority groups indicated: (1) individuals with schizophrenia were more likely than individuals with major depression to engage in both self-violence and violence toward others, and (2) individuals with either disorder were more likely to engage in self-violence than violence toward others. Adjusting for age, for individuals with major depression, participants in all minority groups had significantly higher rates of endorsing self-violence than the U.S. public (*p* < 0.05) while Latina/o and Asian American participants endorsed higher rates of violence toward others than the U.S. public (*p* < 0.05). For individuals with schizophrenia, Asian American participants endorsed significantly lower rates of self-violence and violence toward others than the U.S. public (*p* < 0.05).

### Regressions

Linear regression results (Table 4) indicated that, after controlling for covariates, gender and education showed no significant associations with willingness to seek treatment for any minority group whereas age (*β* = 0.22, *p* < 0.01) was associated with treatment-seeking willingness for Asian American participants. For Black participants, perceiving treatment as likely to improve the disorder was independently associated with greater willingness to seek treatment (*p* < 0.01) while desiring greater social distance (*p* < 0.01) and perceiving individuals with mental disorders as inferior (*p* < 0.05) was associated with reduced willingness. For Latina/o participants, perceiving treatment as likely to improve the disorder was independently associated with greater willingness to seek treatment (*p* < 0.05) while: (1) attributing mental illness to sociomoral causes and God’s will, and (2) perceiving individuals with mental disorders as lower status associated with reduced willingness (*p* < 0.05). For Asian American participants, perceiving greater likelihood for self-violence independently associated with greater willingness to seek treatment (*p* < 0.05) while desiring greater social distance and perceiving individuals with mental disorders as lower status associated with reduced willingness (*p* < 0.05).

## Discussion

Guided by a novel empirical model linking stigma with treatment seeking in racial minority populations, this study was the first to our knowledge to characterize mental illness stigma and its social-cultural contexts in minority Black, Latina/o, and Asian American adults. Pivotally, study findings uncovered the presence of powerful stigmatizing contexts likely to suppress treatment seeking and influence treatment disparities in all three minority groups.

To review the core tenets undergirding this study, to characterize the stigma contexts of these groups, our model proposed that compared to the U.S. public, racial minority individuals living in stigma contexts that suppress treatment seeking might display several distinguishing patterns. First, they could perceive mental disorders as not serious conditions, more likely to improve on its own, and less likely to improve with treatment. Second, they could perceive mental disorders as less likely to be caused by neurobiological factors and endorse individuals with mental disorders as having lower/inferior social status. Third, they could perceive individuals with mental disorders to be more dangerous and desire greater social distancing from these individuals than the U.S. public.

In accordance with our model, Black, Latina/o, and Asian American participants displayed patterns of stigmatizing beliefs/attitudes indicative of unfavorable stigma contexts for treatment seeking. First, compared to the U.S. public, significantly greater percentages of Black, Latina/o, and Asian American participants perceived major depression and schizophrenia as not serious problems, more likely to improve on its own, and less likely to improve with treatment. Over half of participants also believed cultural treatments (e.g., herbal medicines, spiritual rituals) would effectively treat these disorders.

Second, significantly lower percentages of Black and Asian American—but not Latina/o—participants attributed major depression and schizophrenia to neurobiological causes than the U.S. public, with 4 out of 10 participants further endorsing individuals with mental illness as possessing lower social status than, and being inferior to, individuals without mental illness. Black and Latina/o participants also attributed mental disorders to God’s will at over two times the U.S. rates.

Third, significantly higher percentages of Black, Latina/o, and Asian American participants desired greater social distancing from individuals with major depression than the U.S. public and displayed greater rates of unwillingness to engage with these persons across multiple social situations. However, participants generally displayed similar rates of unwillingness to engage with individuals with schizophrenia as the U.S. public.

When separately examining each minority group’s distinct stigma contexts, regression analyses indicated that different stigma components influenced willingness to seek treatment differently across racial minority groups. For Black participants, perceiving treatment as improving mental illness and desiring greater social distance from individuals with mental illness independently predicted willingness to seek treatment. For Latina/o participants, causal attributions such as perceiving mental illness as caused by sociomoral factors (e.g., bad character) or God’s will predicted lower willingness to seek treatment while perceiving treatment as improving mental illness predicted greater willingness. For Asian American participants, perceiving individuals with mental illness as potentially self-violent and having lower status, plus desiring greater social distance from these individuals, predicted lower willingness to seek treatment.

Collectively, study results contribute to the stigma literature by suggesting that while participants in each racial minority group reported more powerful mental illness stigma contexts compared to the U.S. public, the nature of these contexts differed for each minority group. Importantly, these results closely align with prior theories from other notable stigma investigators. For instance, in Yang et al.’s seminal work integrating culture with stigma [6], which was influenced by established theories on moral experience and stigma [42, 57, 58], the researchers noted that stigma’s negative effects and perceptions emerge when a stigmatized condition (e.g., mental illness) threatens what is most at stake in individuals’ local cultural world (i.e., their moral experience) [6]. Specifically, when seeking to understand the influence of stigma and culture on mental health treatment seeking in this study, stigma is expected to affect behaviors such as treatment seeking by threatening the aspects of daily/social life most fundamentally at stake—i.e., “what matters most”—in people’s lives (e.g., social status, relationships, employment), with stigma impinging upon different aspects of daily life based on a group’s valued cultural mores [6, 37, 41, 58, 59]. Consequently, different cultural groups can be theorized to experience slightly different stigma processes/contexts in accordance with their unique cultural values/norms [6, 42, 57]; aligning with our data indicating the presence of differences in the stigma contexts of Black, Latina/o, and Asian American adults in the U.S.

With regard to the specific cultural values/norms of each minority group that contribute to the discovered differences in their stigma contexts, as this is one of the first known studies to assess the unique stigma factors that may suppress treatment seeking in these three minority groups, future in-depth qualitative research is needed to identify the specific cultural elements and mechanisms that may account for both the shared and divergent features of each group’s distinct stigma contexts. For instance, our finding that God’s will predicted willingness to seek treatment in Latina/o but not Black and Asian American adults despite Black and Latina/o adults nationally reporting the highest rates of all U.S. racial groups of (1) being certain that God exists at 94% and 85%, respectively, (67% for Asian Americans) [60], and (2) endorsing religion as “very important” in their lives at 75% and 59%, respectively (36% for Asian Americans) [60] may suggest that Latina/o adults’ religious-informed cultural beliefs around mental illness and mental health treatment may qualitatively differ from Black and Asian American adults in crucial ways that warrant further study. As a possible example, this may involve Latina/o adults being instructed to rely on prayer to treat mental illness symptoms more than members of the other minority groups; thereby reducing Latina/o treatment seeking. Similarly, for Asian Americans, our finding that perceiving individuals with mental illness as possessing lower social status predicted lower treatment-seeking willingness may align with existing research showing that in many Asian cultures, upholding one’s social status (i.e.,”keeping/saving face”) is a core cultural dynamic that may cause Asian adults to fear disclosing mental illness to avoid experiencing ‘social death’ in their communities [42, 61–63]. With regard to stigma commonalities, all three minority groups demonstrated more unfavorable stigma contexts for treatment seeking than the U.S. public. As a possible explanation, Misra et al. [41] noted that all three minority groups share distinctly non-Western cultural values/interpretations that may diverge from the Western biomedical model underlying current treatment services [64]; possibly building mistrust and hesitancy in participants from our three minority groups toward Western treatment services, which may not be oriented to possible non-Western cultural conceptualizations of mental health and illness that may exist in these groups [65].

With regard to clinical implications for programs and interventions to combat the stigma of mental illness, our study findings that different stigma contexts influence treatment seeking across minority groups suggest that intervening to reduce stigma and improve treatment seeking in Black, Latina/o, and Asian American groups may require developing programs/interventions that challenge each group’s unique “*stigma processes that threaten what makes life matter*” [6]. This may include reducing desired social distance among Black adults, stigmatizing attributions in Latina/o adults, and perceptions of dangerousness and lower social status in Asian American adults.

For designing these programs, prior research reveals the importance of developing programs specifically tailored to reduce stigma and enhance community support for treatment services and help seeking [66]. For example, one avenue identified by an expert U.S. forum was to develop narratives combining personal stories with depictions of structural barriers to accessing treatment to increase public support for mental health treatment [66]. In particular, contact-based educational programs that use narratives such as testimonies or fictional stories to increase participant knowledge and exposure to individuals with mental illness have been shown to be effective in reducing stigma [39, 67–69]. Importantly, these contact-based programs may prove especially fruitful with racial minority populations as one study found that Latina/o and Asian American adults exhibited greater decreases on a range of stigma outcomes relative to White adults after completing a contact-based educational program [70]. However, our findings also suggest that due to the potential presence of differences in their stigma contexts, researchers may need to tailor anti-stigma narratives and programs for maximal efficacy with Black, Latina/o, and Asian Americans such as by adapting narratives to feature culturally resonant characters to enhance audience identification and change [71]. Future research should therefore focus on developing culturally tailored narratives and programs that intentionally target/combat the key stigma components shown to influence treatment seeking willingness for each minority group including sociomoral attributions for Latina/o adults and perceptions of lower status and higher self-violence associated with mental illness for Asian American adults.

In addition, future studies should explore the intersecting marginalizations that occur between culture, stigma, and other systems of prejudice and discrimination (e.g., structural racism, classism) [72] affecting racial minority individuals that may influence their stigma experiences/contexts and treatment seeking. Finally, future research should seek to test and refine this study’s novel model, particularly with regard to assessing other key factors that may play an important role in influencing stigma contexts and perceptions in racial minority groups. This includes examining potentially key geographic, environmental, and cultural conditions such as differences between urban and rural communities, community access to mental health treatment services, as well as gender, education, religious beliefs, physical disability, and immigration status to further enrich our understanding of how cultural and community factors may influence stigma contexts and treatment seeking in racial minorities.

Several study limitations exist including use of cross-sectional data, which prohibited evaluating causation between our stigma and treatment-seeking variables. Similarly, the single item, dichotomous nature of the mental health treatment willingness variable may have limited variance in the regression models. Thus, future researchers may wish to include additional treatment items or scales to more comprehensively explore these relationships. Further, although using Qualtrics research panels had numerous advantages in this study vs. traditional recruitment methods by allowing for the effective recruitment of minority participants via quota sampling based on participants’ demographic characteristics [48], limitations include a lack of information involving the (1) precise size, number, and demographic breakdown of the diverse participant panels used, (2) response rate across the multiple panels used, and (3) participants’ identifying information, which prevented follow-up or longitudinal studies by the investigators. Also, while we assessed stigma in the three largest U.S. racial minority groups, we did not assess other important minority groups such as Indigenous and sexual and gender minorities; necessitating further investigations with other minority groups to refine our model. Additionally, as mental health issues are rising in younger populations, researchers should study the stigma contexts of youth/young adults to bolster treatment seeking in this at-risk population [73].

## Conclusion

Data from this novel study of understudied racial minority adults revealed the existence of strong stigma contexts among Black, Latina/o, and Asian Americans, with stark contextual differences noted between these groups as different stigma components differentially associated with treatment seeking. These differences: (1) confirm the importance of oversampling racially diverse populations in stigma research to identify racial/cultural variations in stigma, broadening our understanding of key stigma mechanisms/pathways such as divergent cultural conceptualizations of mental illness, and (2) suggest that different groups may require tailored interventions to address “*what matters most*” within their unique stigma contexts. Finally, although this study represents a positive step in characterizing mental illness stigma in understudied minority groups, further research is needed to tailor anti-stigma programs/interventions to bolster minority treatment seeking; thereby addressing a potentially important driver of mental health disparities affecting racial minority communities.

## Figures and Tables

**Table 1:** Characteristics and descriptive statistics for sample participants (*N* = 614)

Variables	% (# of cases)
Gender	
Female	47.2 (290)
Education	
< High school	8.8 (52)
High school	39.6 (191)
Some college	27.7 (170)
≥ College	30.1 (185)
Marital Status	
Single/Separated/Divorced	68.1 (418)
Married/Living as married	31.9 (196)
Race/Ethnicity	
Black	33.1 (203)
Latina/o	32.9 (202)
Asian	31.4 (193)
	(Mean ± SD)
Age (Years)	34.78 ± 14.68

**Table 2:** Perceived seriousness, treatability, and causes of major depressive disorder and schizophrenia for study sample vs. 2018 GSS U.S. national sample

Perceived Cause	Black Sample (Vignette, %)		Latina/o Sample (Vignette, %)		Asian Sample (Vignette, %)		U.S. GSS Sample (Vignette, %)	
	MDD	Schiz	MDD	Schiz	MDD	Schiz	MDD	Schiz
Treatment								
Serious problem	87.8	**87.8**	**85.3**	**85.4**	**83.9**	92.5	95.4	97.8
Improve on own	53.3	**50.0**	**56.9**	**47.9**	50.5	**45.2**	33.6	18.3
Improve with treatment	**83.3**	**81.6**	**78.4**	**84.4**	88.2	**88.2**	95.4	96.4
Improve with cultural treatment	57.8	63.3	52.0	53.1	49.5	50.5	–	–
Neurobiological Attributions								
Mental illness	66.7	**70.4**	64.7	**82.3**	65.6	**76.3**	77.5	94.2
Chemical imbalance in the brain	**52.2**	**70.4**	**53.9**	**76.0**	**58.1**	**72.0**	82.2	93.5
Genetic or inherited problem	**53.3**	**58.2**	**51.0**	67.7	**41.9**	**52.7**	66.2	76.1
Neurobiological conception (2-item)	**73.3**	**82.7**	74.5	89.6	**71.0**	**81.7**	86.9	94.6
Social or Moral Attributions								
Own bad character	43.3	39.8	43.1	44.8	36.6	44.1	35.0	37.8
Way person was raised	44.4	52.0	56.9	52.1	45.2	**59.1**	45.2	43.6
Sociomoral conception (2-item)	61.1	65.3	70.6	65.6	62.4	71.0	55.8	59.5
God’s will	**42.2**	**44.9**	**36.3**	**41.7**	26.9	28.0	14.3	18.1
Life stress	**74.4**	**74.5**	**82.3**	79.2	88.1	77.4	94.1	85.2
Mental Illness Status								
Lower Status (Fair)	41.1	44.9	40.2	40.6	45.2	48.4	–	–
Inferior (Accurate)	50.0	44.9	43.1	42.7	40.9	45.2	–	–

GSS = General Social Survey; MDD = Major depressive disorder; Schiz. = Schizophrenia

Boldface indicates the percentage for the group significantly differed from the percentage for the U.S. public (*p* < 0.05)

**Table 3:** Stigma as perceived dangerousness and social distance for study sample vs. 2018 GSS U.S. national sample

	Black Sample		Latina/o Sample		Asian Sample		U.S. GSS Sample	
Vignette: Major Depressive Disorder	Mean ± SD	% Unwilling	Mean ± SD	% Unwilling	Mean ± SD	% Unwilling	Mean ± SD	% Unwilling
Social Distance (5-item)	2.31 ± .65	**40.0**	2.18 ± 2.04	**25.5**	2.28 ± .64	**31.2**	2.44 ± 1.56	20.2
Unwilling to move next door	2.16 ± .82	**28.9**	2.20 ± .96	**35.3**	2.19 ± .80	**28.0**	2.43 ± 1.80	18.3
Unwilling to socialize	2.39 ± .90	**42.2**	2.12 ± .95	**31.4**	2.16 ± .88	**32.3**	2.30 ± 1.70	18.5
Unwilling to make friends	2.18 ± .86	**36.7**	1.99 ± .94	**29.4**	2.10 ± .85	**25.8**	2.17 ± 1.65	12.9
Unwilling to work closely on a job	2.30 ± .83	**40.0**	2.13 ± .98	33.3	2.19 ± .84	31.2	2.51 ± 1.63	29.6
Unwilling to have person marry into family	2.51 ± .94	48.9	2.46 ± .95	**45.1**	2.76 ± .85	**62.4**	2.80 ± 1.82	41.7
	Mean ± SD	% Likely	Mean ± SD	% Likely	Mean ± SD	% Likely	Mean ± SD	% Likely
Danger to others	2.53 ± 1.01	46.7	2.46 ± .92	**50.0**	2.53 ± .84	**46.2**	2.61 ± 1.65	33.8
Danger to self	2.19 ± .90	**64.4**	2.16 ± .87	**73.5**	2.23 ± .63	**71.0**	3.29 ± 1.49	82.4
Vignette: Schizophrenia								
Social Distance (5-item)	2.24 ± .66	**36.7**	2.31 ± 2.19	**38.5**	2.58 ± .08	49.5	2.91 ± 1.16	59.8
Unwilling to move next door	2.27 ± .89	41.8	2.27 ± .81	**37.5**	2.46 ± .98	47.3	2.90 ± 1.62	48.1
Unwilling to socialize	2.21 ± .88	36.7	2.32 ± .84	43.8	2.55 ± .95	52.7	2.74 ± 1.41	45.7
Unwilling to make friends	2.03 ± .84	**22.4**	2.08 ± .71	25.0	2.43 ± .95	40.9	2.55 ± 1.41	34.8
Unwilling to work closely on a job	2.17 ± .93	**32.7**	2.18 ± .85	**37.5**	2.45 ± .97	**43.0**	3.07 ± 1.30	68.3
Unwilling to have person marry into family	2.54 ± .95	56.1	2.71 ± .92	60.4	3.03 ± .83	72.0	3.28 ± 1.46	70.5
	Mean ± SD	% Likely	Mean ± SD	% Likely		% Likely	Mean ± SD	% Likely
Danger to others	2.18 ± .85	65.3	2.28 ± .76	61.5	2.34 ± .88	**55.9**	3.15 ± 1.50	69.3
Danger to self	1.86 ± .79	83.7	1.81 ± .69	88.5	2.03 ± .77	**75.3**	3.64 ± 1.36	91.6

Percentages represent combined “definitely unwilling” and “probably unwilling” or “very likely” and “somewhat likely” responses

GSS = General Social Survey; MD = Major depression; Schiz. = Schizophrenia

Boldface indicates the percentage for the group significantly differed from the percentage for the U.S. public (*p* < 0.05)

**Table 4:** Mental health treatment willingness predicted by mental illness attributions, status, seriousness, treatability, social distance, and dangerousness

DV = Mental Health Treatment Willingness					
BLACK					
Step	Predictor	*β*	*b* (*SE*)	*F*	*R*^2^ (Δ*R*^*2*^)
1: *Seriousness & Treatability*	Serious problem	−.06	−.07 (.10)	2.68*	.12*
	Improve on own	−.02	−.01 (.08)		
	Improve with treatment	**.25** **	.28** (.10)**		
2: *Attributions & Status*	Mental illness	.01	.01 (.09)	2.11*	.16* (.04)*
	Sociomoral conception	.02	.05 (.14)		
	Neurobiol. conception	.03	.07 (.17)		
	God’s will	.02	.02 (.07)		
	Lower status	−.02	−.05 (.15)		
	Inferior	**−.21** *	**−.37 (.15)** *		
3: *Stigma Backbone*	Social distance	**−.22** **	−.30** (.10)**	2.35*	.20* (.04)*
	Danger to others	−.08	−.08 (.08)		
	Danger to self	.04	.04 (.09)		
LATINA/O					
Step	Predictor	*β*	*b* (*SE*)	*F*	*R*^2^ (Δ*R*^*2*^)
1: *Seriousness & Treatability*	Serious problem	−.02	−.02 (.10)	2.20*	.10*
	Improve on own	−.05	−.05 (.09)		
	Improve with treatment	**.21** *	.23* (.09)*		
2: *Attributions & Status*	Mental illness	−.05	−.06 (.09)	2.30*	.16* (.06)*
	Sociomoral conception	**−.16** *	−.32* (.16)*		
	Neurobiol. conception	.01	.03 (.19)		
	God’s will	**−.18** *	−.16* (.08)*		
	Lower status	**.17** *	.31* (.15)*		
	Inferior	.01	.02 (.16)		
3: *Stigma Backbone*	Social distance (5-item)	.08	.11 (.11)	1.96*	.17* (.01)*
	Danger to others	.02	.02 (.10)		
	Danger to self	−.06	−.07 (.10)		
**ASIAN**					
1: *Seriousness & Treatability*	Serious problem	−.12	−.15 (.12)	2.05*	.10*
	Improve on own	−.01	−.01 (.08)		
	Improve with treatment	.02	.02 (.10)		
2: *Attributions & Status*	Mental illness	.05	.05 (.09)	1.81*	.14* (.04)*
	Sociomoral conception	.10	.18 (.16)		
	Neurobiol. conception	.09	.18 (.16)		
	God’s will	−.01	−.01 (.07)		
	Lower status	**−.17** *	**−.30 (.15)** *		
	Inferior	.09	.15 (.15)		
3: *Stigma Backbone*	Social distance (5-item)	**−.18** *	−.21* (.10)*	2.29*	.21* (.07)*
	Danger to others	−.12	−.12 (.10)		
	Danger to self	**.18** *	−.22* (.11)*		

* *p* < .05;

** *p* < .01

Demographics = age, gender, education, vignette completed

Neurobiol. = neurobiological

No demographics were significant except for age (*β* = .22, p < .05) and vignette completed (*β* = −.19, p < .05) for Asian Americans
